# ‘Small volume—big problem’: culturing *Yarrowia lipolytica* in high-throughput micro-formats

**DOI:** 10.1186/s12934-024-02465-3

**Published:** 2024-06-24

**Authors:** Ewelina Celińska, Maria Gorczyca

**Affiliations:** Department of Biotechnology and Food Microbiology, Poznan University of Life Sciences, 60‑637 Poznań, Poland

**Keywords:** High-throughput screens, Yeast, *Yarrowia lipolytica*, Microfluidics, Micro-titer-plates, Droplet sorting, Square plates, Screening protocol

## Abstract

With the current progress in the ‘design’ and ‘build’ stages of the ‘design-build-test-learn’ cycle, many synthetic biology projects become ‘test-limited’. Advances in the parallelization of microbes cultivations are of great aid, however, for many species down-scaling leaves a metabolic footprint. *Yarrowia lipolytica* is one such demanding yeast species, for which scaling-down inevitably leads to perturbations in phenotype development. Strictly aerobic metabolism, propensity for filamentation and adhesion to hydrophobic surfaces, spontaneous flocculation, and high acidification of media are just several characteristics that make the transfer of the micro-scale protocols developed for the other microbial species very challenging in this case. It is well recognized that without additional ‘personalized’ optimization, either MTP-based or single-cell-based protocols are useless for accurate studies of *Y. lipolytica* phenotypes. This review summarizes the progress in the scaling-down and parallelization of *Y. lipolytica* cultures, highlighting the challenges that occur most frequently and strategies for their overcoming. The problem of *Y. lipolytica* cultures down-scaling is illustrated by calculating the costs of micro-cultivations, and determining the unintentionally introduced, thus uncontrolled, variables. The key research into culturing *Y. lipolytica* in various MTP formats and micro- and pico-bioreactors is discussed. Own recently developed and carefully pre-optimized high-throughput cultivation protocol is presented, alongside the details from the optimization stage. We hope that this work will serve as a practical guide for those working with *Y. lipolytica* high-throughput screens.

**Supplementary Information:**

The online version contains supplementary material available at 10.1186/s12934-024-02465-3.

## Introduction

Every synthetic biology project, to avoid bottlenecks, requires fine-tuning the ‘design-build-test-learn’ cycle at all stages. With the current advent of computational design tools, DNA synthesis capacity, and genome editing technology, a high fraction of synthetic biology projects become ‘test-limited’ [8]. Advances in the parallelization of cell population cultivations by the use of microtiter plates (MTPs) or macro-plates combined with robotic handling and automatic data acquisition are of great aid [4, 41, 45, 58]. Likewise, the progress in single-cell-based flow cytometry approaches, like fluorescence-assisted cell sorting (FACS) and fluorescence-assisted droplet sorting (FADS), debottlenecks the ‘test’ stage in specific applications [5, 15, 35, 57, 62].

For some microbial species, the metabolic footprint left by down-scaling to microvolumes is negligible. For example, a good quantitative agreement in the performance of *Escherichia coli* and *Acinetobacter* grown on a microwell scale (1 mL) and in a laboratory stirred-tank bioreactor (2 L) [21] was reported. Highly similar patterns of growth were also found for several laboratory strains of *Saccharomyces cerevisiae* (including CEN‐PK.2) cultured on a ‘micro’ scale (0.35 mL) and a ‘medium’ scale of 10 mL [58]. Maximum growth rates and biomass accumulation of *S. cerevisiae* cells grown in MTPs for ‘microvinification’ were indistinguishable from those observed in self-induced anaerobic flask cultures [42]. Yet, in the majority of cases, scaling-down leaves some metabolic footprint on the cell,making the scaling-up or -down a separate and demanding task in bioprocess development. The problem was highlighted *i.a.* by [51], who performed comparative cultures of engineered *Komagataella phaffi* (formerly–*Pichia pastoris* in deep-well plates and 1 L bioreactor. The small-scale cultures disallowed phenotype development and no differences were seen in volumetric enzyme activity between four differently-engineered strains; while the phenotypes were significantly different in the bioreactor cultivations.

*Yarrowia lipolytica*, is one of such demanding species, for which scaling-down inevitably leads to perturbations in phenotype development. Strictly aerobic metabolism, propensity for filamentation and adhesion to hydrophobic surfaces, spontaneous flocculation, and high acidification of media are just several characteristics that make the transfer of the micro-scale protocols developed for the other yeast species very challenging. Our own experience and the experience of our Colleagues (e.g. [5]) provide evidence that without additional consideration and optimization, either MTP-based or single-cell-based protocols are useless for accurate studies of *Y. lipolytica* phenotypes.

This review summarizes the research in parallelization of *Y. lipolytica* culturing, highlighting the pitfalls that occur most frequently. We hope that it will serve as a practical guideline for those working with *Y. lipolytica* high-throughput screens.

## How small can we go with *Yarrowia*? And what are the costs?

**Fig. 1:**
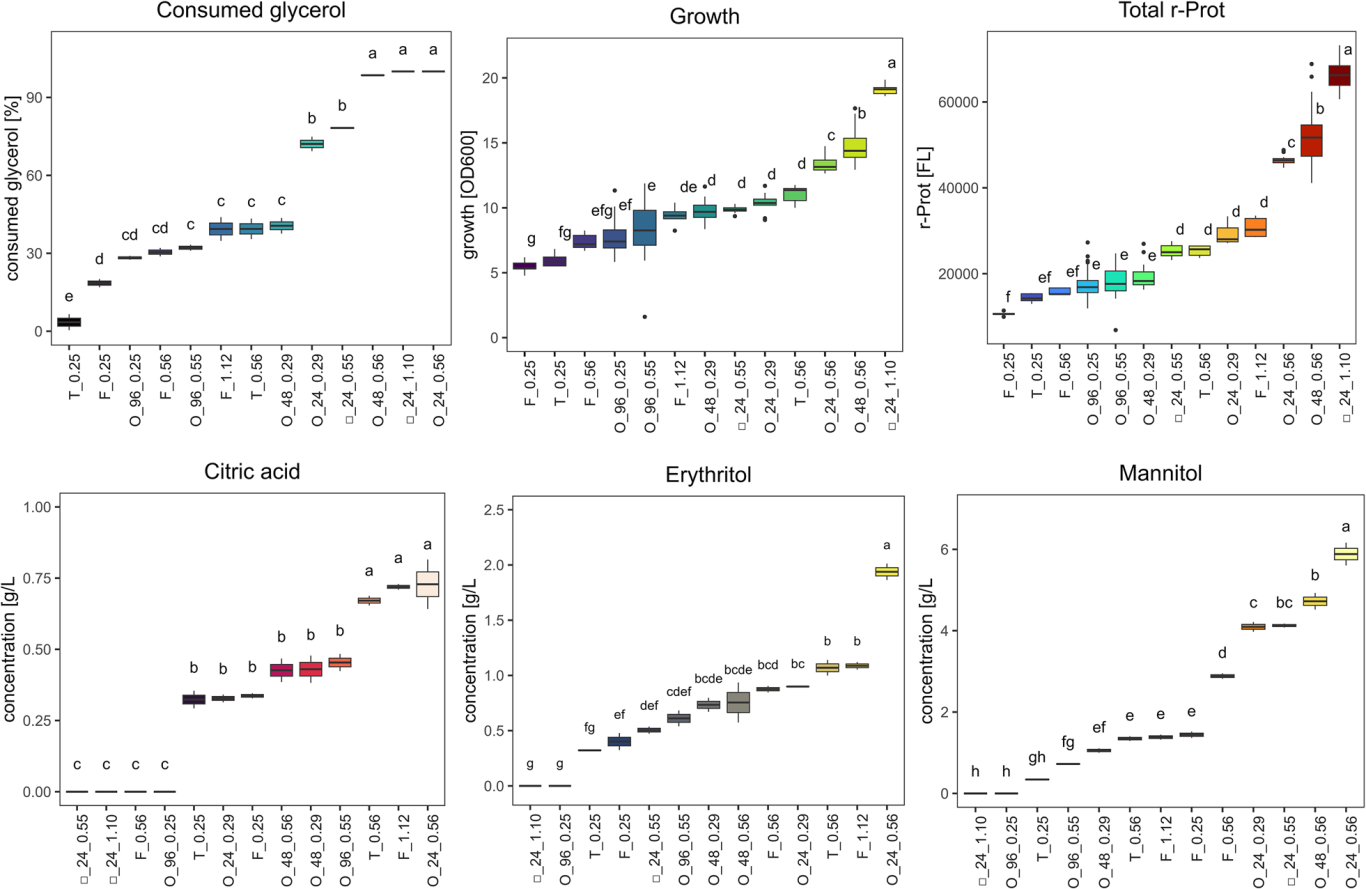
Glycerol (GLY) consumption [%] metabolites concentration (citric acid, CA, erythritol, ERY, and mannitol, MAN), growth [OD600], and fluorescence from an intracellular reporter protein rProt, determined in the post-culturing of *Y. lipolytica* strain run in different scales and vessels (encoded according to Table 1), at different kLa parameter settings (Mendeley data [11]). *Y. lipolytica* strain used in this experiment was JMY2810 (genotype: *MATa, ura3::pTEF-RedStar2-LEU2-Zeta-URA3ex-pTEF-empty, leu2-270, xpr2-322*; phenotype: ΔAEP, ΔAXP, suc + , ura + , leu + , intracellular RedStarII, Zeta platform). The main cultures were continued for 48 h and samples were collected at the end of the cultivation time. Precultures were developed for 18 h at 28 °C in the 300 mL shake flasks. The main cultures were inoculated at 5% (v/v). Samples were analyzed for growth and fluorescence from the reporter protein (RedStar2) following dilution in 0.75% NaCl (POCH) to match a linear range of the methods. Absorbance was measured at 600 nm in transparent 96-well plates (Costar; Merck). FL was determined under at ex/em 550/595 nm in black opaque plates (Thermo Fisher Scientific). Both measurements were done using a Tecan Spark automatic plate reader (Tecan Group Ltd., Mannedorf, Switzerland). pH level at the end of cultures was determined in the supernatant using indicatory strips (POCh, Poland). Each variant of the flask (F) and tube (T) cultures was conducted in pentaplicate, cultures in 96-well 48-well, and 24-well were conducted in 48, 24, and 12 parallel runs. Values show the average mean from the replicates. Error bars show ± SD from: (i) pentaplicate for flask and tube cultures, (ii) 48, (iii) 24, or (iv) 12 parallel runs of the cultures run in 96-well 48-well, and 24-well, respectively. Letters indicate homogenous groups determined in statistical analysis. Statistical significance of the difference in a given measure was assessed by analysis of variance (ANOVA) test, with a significance level set at p-value < 0.05 (RStudio and Visual Studio Code, Microsoft), and equality of variances was checked with the Levene test. The homogenous groups were calculated using Tukey HSD post-hoc analysis (RStudio with relevant packages)

To quantitatively describe this problem, we previously conducted a small ‘proof-of-concept’ experiment by culturing *Y. lipolytica* strain synthesizing a fluorescent reporter protein (rProt) in different volumes ([11], Table 1). Growth and fluorescence from the intracellular rProt, as well as substrate and metabolite concentration, were analyzed (Fig. 1). For all the thirteen variants we calculated a volumetric mass transfer coefficient (k_L_a), according to Eq. 1 [43, 44]:

1 $${k}_{L}a=6.67\times {10}^{-6}\times {n}^{1.16}\times {V}_{L}^{-0.83}\times {d}_{0}^{0.38}\times {d}^{1.92}$$

where, vessel diameter (d), culture volume (V_L_), shaking frequency (n), shaking amplitude (d_0_).

**Table 1:** Technical parameters of the compared culturing vessels and conditions for mixing the cultures

Code	Topology/type of vessel	Diameter	Total vol	Culture vol	Filling	Shaking frequency	Shaking amplitude	Calc. K_L_a
	In horizontal plane [mm]		[mL]	[mL]	[%]	[rpm]	[cm]	[/h]
O_96_0.25	○ MTP U-shaped	6.5	0.3	0.2	67	450	0.2	0.25
O_96_0.55	○ MTP U-shaped	6.5	0.3	0.08	27	450	0.2	0.55
O_48_0.29	○ MTP flat-bottom	9.75	1.3	0.5	38	450	0.2	0.29
O_48_0.56	○ MTP flat-bottom	9.75	1.3	0.2	15	450	0.2	0.56
O_24_0.29	○ MTP flat-bottom	15.5	3.3	1	30	380	0.2	0.29
O_24_0.56	○ MTP flat-bottom	15.5	3.3	0.45	14	380	0.2	0.56
□_24_0.55	□ MTP conical-baffled-bottom	17	11	2.5	23	250	1.91	0.55
□_24_1.1	□ MTP conical-baffled-bottom	17	11	1.1	10	250	1.91	1.1
T_0.25	○ tube U-bottom	14	24	2	8	180	1	0.25
T_0.56	○ tube U-bottom	14	24	0.75	3	180	1	0.56
F_0.25	○ Erlenmeyer flask	80	250	150	60	180	2	0.25
F_0.56	○ Erlenmeyer flask	80	250	58	23	180	2	0.56
F_1.12	○ Erlenmeyer flask	80	250	25	10	180	2	1.12

K_L_a is a parameter that determines the rate at which a gaseous compound transfers between the gas and the liquid phases. It has two principal components: the mass transfer coefficient (k_L_) and the specific exchange surface (a). It is impacted by the geometry of the culturing vessel (volume or maximum diameter), shaking parameters (amplitude and frequency), and the liquid phase properties (volume and viscosity). As demonstrated previously, the oxygen transfer rate depends linearly on the culture volume (V_L_), and twice the lower volume V_L_ contributes to twice the higher oxygen transfer rate [18]. Parameters such as temperature and composition of the liquid phase, determining solubility and diffusion of oxygen, specifically affect the k_L_ component. As evidenced earlier [46], minor changes in liquid viscosity caused by the uptake of nutrients and the formation of cellular biomass can be ignored. K_L_a is commonly used as a proxy of ‘aeration rate’ in bioprocessing, and a useful tool when re-scaling the process. Equation 1 was developed for standard glass Erlenmeyer flasks with hydrophilic walls, shaking frequencies of 50–500 rpm, relative filling volumes of 4–20% (the relative filling volume is defined as the filling volume divided by the nominal flask volume), shaking diameters of 1.25–10 cm, and nominal flask volume between 50 and 1000 mL [43, 44, 50]. The ‘proof-of-concept’ experiment [11] covered vessels beyond those indicated (in volume and geometry), so the use of Eq. 1 for modeling kLa in such a wide set of different vessels is burdened with uncertainty. Nevertheless, estimations of the impact of relative culture volume change or culturing system format on the relative change in oxygen availability (expressed in kLa or oxygen transfer velocity, OTV; Table S1) show good agreement between different studies [11, 16, 18, 26, 29, 59]. For example, for a culture run in O_48-well format, a 40% decrease in volume caused an increase in kLa by 63% in a study by [29], while decreasing the volume by 60% led to an increase in kLa parameter by 93% in the ‘proof-of-concept’ experiment [11]. Changing the culturing format from O_96-well to O_24-well triggered an increase in the kLa parameter by 9% [16] or 2% [11]. Going down from a test tube culture to O_96-well decreased the kLa level by 46% [59], or 54% [11]. More details on the specific culturing system can be found in Table S1 (Supplementary Material). Still, the kLa values in Table 1 calculated according to Eq. 1 should be perceived as only rough estimations.

As can be inferred from Fig. 1 [11], consumed glycerol (GLY) and growth, but also amounts of rProt, and the principal metabolites (citric acid, CA, erythritol, ERY, and mannitol, MAN) were in high positive correlation (consumed GLY and growth, r = 0.85 across all the conditions). The highest values for growth and GLY consumption were reported for cultures run in 24-well plates, either round (O)- or square (□)-shaped, and in an O-shaped 48-well plate, but in this case—only when k_L_a was set at 0.56. Comparison of cultures run at different k_L_a in a specific vessel suggests a straightforward impact of k_L_a level on GLY consumption, rProt amounts, and the amounts of the major metabolites. Only the case of □_24_1.1 escaped this rule, as no metabolites were found in the post-culturing medium. The lack of metabolites in the post-culturing medium in our experiment could be a consequence of either very robust (□_24_1.1) or very limited (O_96_0.25) metabolism. To our interpretation, in cultures □_24_1.1 *Y. lipolytica* reconsumed CA, ERY, and MAN, which served as an additional carbon source. The highest biomass accumulation in this specific case supports this statement. In other words, the actual amounts of oxygen supplied in □_24_1.1 surpassed those provided in tubes, flasks, 48-well, and 96-well MTPs, enabling oxygenation of all GLY from the medium, as well as the reutilization of own metabolites. This notion complies with a general rule saying that in aerobic metabolism the consumption of the substrate is positively correlated with the oxygen supply. However, when comparing the correlated parameters (e.g. GLY consumption) achieved at the same k_L_a values but in different vessels, this correlation is no longer valid; for example, at k_L_a of 0.55- 0.56, GLY was utilized at 30% (O_96_0.55) to 100% (O_24_0.56). Correspondingly, GLY consumption degree could reach from 3.4% (T_0.25) to over 70% (O_24_0.29) at the estimated k_L_a of ~ 0.27. This notion means that either the calculated k_L_a values are not adequate measures of oxygen availability in our experimental setup, or that the scaling between selected vessels is subjected to some non-considered ‘indirect factors’. Several significant ‘indirect factors’ affecting such a vessel-to-vessel variation were identified and well-evidenced (discussed hereafter). In our ‘proof-of-concept’ experiment, the importance of those ‘indirect factors’ (not covered by Eq. 1) is particularly well seen under the limited oxygen supply. For k_L_a set at ~ 0.27, the GLY consumption (or growth) could reach one of four (or three) significantly different levels, represented in separate homogenous groups (Fig. 1; [11]). On the other hand, the adopted k_L_a model [43, 44] does not consider, for example, the head-space volume (partly, but not perfectly expressed as % filling in Table 1). The mass transfer between the head-space and the bulk of the culture is facilitated when compared with the mass transfer across the cover of any kind, acting as a mechanical hindrance. Its direct effect could be reliably assessed in cultures of the same working volume, with the same effective mass transfer area, but differing in the headspace volume (no such condition was studied here).

**Fig. 2:**
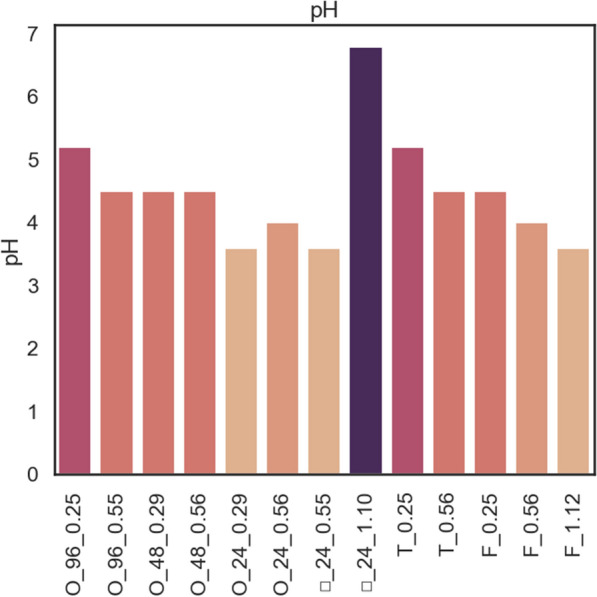
pH level determined in the culture medium supernatants of *Y. lipolytica* strain run in different scales and vessels (encoded according to Table 1), at different kLa parameter settings (Mendeley data [11])

In our accompanying ‘proof-of-concept’ experiment the synthesis of metabolites was concomitant with changes in the medium acidity (Fig. 2; [11]). Significant changes in this parameter (> 0.5 unit) were observed in the 24-well MTP cultures and flasks with k_L_a > 0.56. The unusual increase in the pH value in □_24-well cultures is a known phenomenon occurring at the end of *Y. lipolytica*, highlighting cell death [9]. This minor observation on varying pH depending on the culturing vessel, highlights an issue of technical importance. Namely, depending on the growth conditions and aeration rate, the cell population may yield products of different oxidation levels (for *Y. lipolytica*–CO_2_ or organic acids/polyols). The variation in the product profile impacts medium acidity, and hence—requires different technical solutions to stabilize this parameter.

**Fig. 3:**
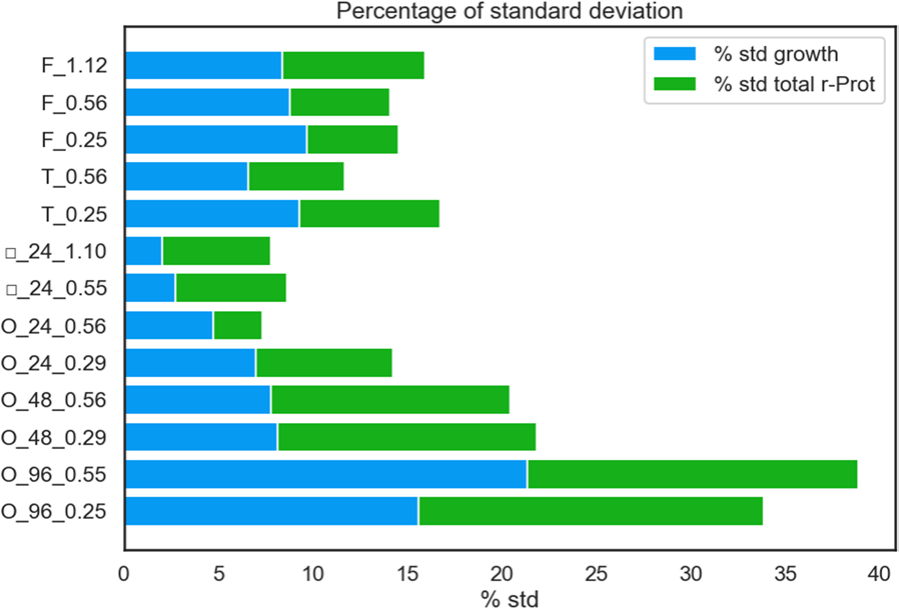
Variability of data on growth and rProt amounts read in *Y. lipolytica* strain cultures run in different scales and vessels (encoded according to Table 1), at different kLa parameter settings (Mendeley data [11]). Variability is expressed as a share of standard deviation in the mean result obtained from a specific culture variant. Each variant of the flask and tube cultures was conducted in pentaplicate, cultures in 96-well 48-well, and 24-well were conducted in 48, 24, and 12 parallel runs

To assess reproducibility between repetitions of a specific culture variant, we calculated the percentage of standard deviation within each result for growth and rProt (Fig. 3). The least consistent results were obtained in cultures run in 96-well plates (%SD reached approx. 15–20%), followed by cultures run in test tubes (%SD reached approx. 5–10%). Surprisingly, the least variable were the repetitions of cultures conducted in 24-well plates (and not in the flasks, as presumed). In these cases, the percentage share of SD in the result reached several % (%SD between 2 and 7%). Growth readouts were the most consistent in the cultures run in □_24-well plates (%SD 2–2.7%). The reproducibility of cultures run in these vessels was assessed previously for bacterial cultures (mainly *Pseudomonas* and *Rhodococcus* spp) [18]. For the majority of strains, the duplicates differed by less than 5%. Depending on the strain, the difference between the replicates could reach more (up to 20%).

Collectively, the ‘proof-of-concept’ experiment showed that going below the 24-well plate with *Y. lipolytica* cultures is burdened with greatly increased well-to-well variability (Fig. 3; the smaller the volume, the bigger the SD). Cultures in 96-well plates seem to be inadequate for *Y. lipolytica* culturing, at least when a comparison of different phenotypes is aimed at, and not just revival of a strain or biomass propagation. Noteworthy, our experiment demonstrated that even for such a ‘demanding’ species, cultures run in 24-well plates constitute a mature alternative to Erlenmeyer flasks; offering high reproducibility, efficient substrate consumption, growth, and rProt synthesis. This format also greatly helps to make the handling and screening of a large number of strains less time- and material-consuming (the □-plates are reusable). The orbital shaking used for 24-well MTPs was suitable to generate sufficient oxygen provision, even surpassing the ones achieved in Erlenmeyer flasks.

## Mechanistic view on scaling-down and the main limitations for *Y. lipolytica* culturing

### Multi-cell population culturing – MTPs

The phenomena taking place upon down-scaling of microbial bioprocesses to MTPs were thoroughly investigated and discussed in a series of works [17–20]. In this review, we focus on volumes that could be useful in high-throughput screens. In such a case, a cell of (average) several μm is cultured either in the liquid bulk of several mm (MTPs) or cm (flasks). As stated by [17] such a dimensional change in the culturing vessel has a negligible impact on the cell’s physiology.

The principal limitations of (*Y. lipolytica*) micro-volume culturing in MTPs are insufficient aeration rates and small working volumes [18]. The former becomes specifically troublesome for microbes with high oxygen demands, like *Y. lipolytica*. Typically, limited aeration is overcome by increasing mixing frequency and amplitude, but on the other side—it leads to increased risk of cross-contaminations between the wells. Interestingly, these apparent limitations are also shaped by other ‘indirect factors’ affecting cellular physiology, and gaining importance upon down-scaling. Awareness of their occurrence is important to develop counteracting measures. These ‘indirect factors’ are (i) the ratio of the effective gas–liquid exchange area to the culture volume (considered in Eq. 1), (ii) the increased importance of the surface tension, which counteracts the flow and movement of the culture bulk in micro-volumes. The latter is specific to MTPs and has not been observed for larger scales [26]. Since the bulk of the medium in MTPs is relatively small, its inertness is small, and the movement induced by the centrifugal force (shaking amplitude) is overbalanced by adhesion (liquid molecule to the vessel) and cohesion (liquid molecules interaction) forces. Adhesion and cohesion forces remain constant irrespectively from the culture volume–they depend on the properties of the liquid and the vessel’s material. So up to a specific shaking intensity, the liquid surface remains horizontal in O-shaped micro-wells of small bulk volumes (no turbulence occurs, and no expansion of the gas–liquid exchange area occurs). Turbulence may be introduced by using square-shaped micro-wells. Then, the corners of the well act as baffles, greatly expanding the effective gas–liquid exchange area, and amplifying the oxygen transfer rate by a factor of 2 [17, 20]. As demonstrated, the surface area is a more important determinant of the oxygen transfer rates than the frequency of shaking [18].

**Fig. 4:**
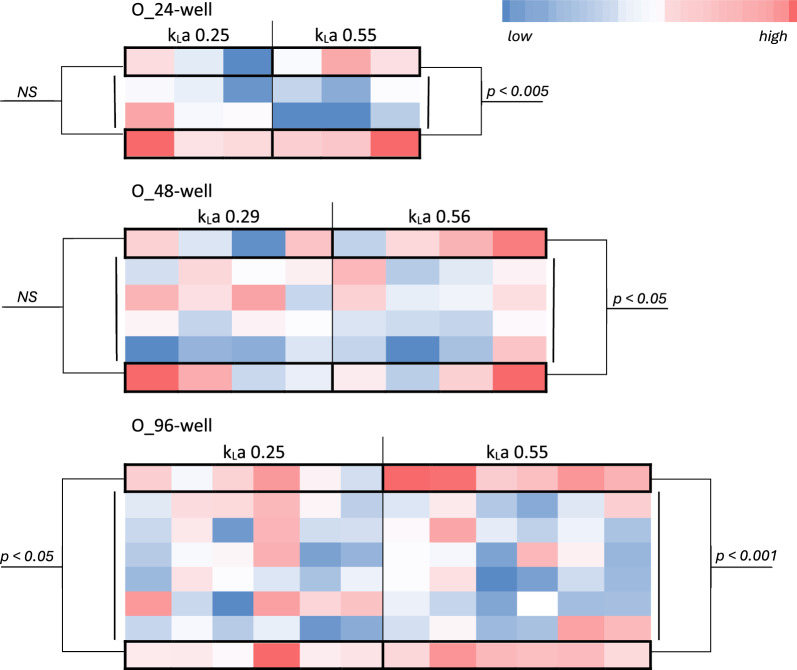
Exemplary heat-maps showing growth of a *Y. lipolytica* strain in typical O-shaped 24-, 48-, and 96-well MTPs with solid cover. Color spaces correspond to well in the MTPs. Growth was measured as absorbance at OD600 (Mendeley data [11]). The values are color-coded according to a legend. The same strain was grown in all the wells, in the same culture medium. Comparisons of the color scale should be done only within a specific section covering identical culture variants. Inter-plates comparisons of the color are not adequate. Statistical significance was determined via ANOVA analysis (Mendeley data [11])

Another significant problem faced in MTP culturing is that the oxygen diffusion rates to an individual well are typically affected by its position in the MTP, with the central wells being less aerated (Fig. 4; [11]). Our experience shows that the problem is valid for 24-, 48-, and 96-well MTPs equipped in a typical solid plastic cover or sealed with ‘breath freely’ film. On the other hand, such an effect was not observed for □_24-well MTPs with individual air exchange systems above each well. In these MTPs, at k_L_a 0.55 and 1.1–the difference between inner and outer (central and peripheral) wells was not significant at p < 0.001. Such an uncontrollable well-to-well variation leads to misinterpretation of the results coming from a single MTP. Interestingly, our previous studies showed, that upon (anoxia) stress *Y. lipolytica* population splits into more and less metabolically active cells, and the latter subpopulation increased in counts with the time of exposure to stress [23, 24], so the heterogeneity came from cell-to-cell variation within a single well, rather than from global better or worse performance of a population at a specific location in the MTP.

Another recognized challenge of MTPs culturing is that insufficient aeration contributes to the differential profile of metabolites being produced in different wells. Consequently, depending on the strain’s biology, difficulties in maintaining stable and comparable pH across the plate may occur; further amplifying the well-to-well variation in MTPs.

And finally, in terms of well-to-well variation illustrated in Fig. 4, the better aeration of the well located at the corners has also a negative side – it contributes to enhanced culture volume loss in those wells. Hence, especially when working with the low-volume 96-well format, peripheral wells are typically used as cell-less humidifiers filled with medium or water, and are lost from the analysis.

To overcome these limitations, the inventors of □_24/96-well plates proposed a cover system with holes above each well, but tightly sealing rants with a soft-silicone mat [17, 18]. Such a solution secures individual air exchange from each location on the plate, and limits the impact of the neighboring wells and cross-contaminations. The wells in the central part of a plate are equally well aerated as those in the peripheries. Indeed, in our ‘proof-of-concept’ experiment, the growth of *Y. lipolytica* in □_24-well plates was not significantly different between central and peripheral wells (p-value < 0.001).

In addition, the implementation of this individual aeration system unified the propensity for volume loss across the plate. Still, thanks to a multi-layer covering system and the selection of the material, the volume losses are negligible (10 μL per day), and cross-contaminations are also eradicated ([18] and own experiments discussed hereafter).

### Single-cell-per-vessel approaches

A further step in scaling-down microbial cultures is miniaturization to pico-liter volume droplets, each initially loaded with a single cell. Such an approach differs from what is typically called ‘single-cell analysis’ by Fluorescence-Assisted Cell Sorting (FACS), which is an analytical method that does not require the droplet-forming step. The pico-liter cultivation followed by the droplet analysis and sorting is called Fluorescence-Assisted Droplet Sorting (FADS) [2].

An individual microbial cell can be entrapped in a ‘single-cell vessel’ either by using agarose beads or by creating an emulsion. A microbial cell must always be suspended in the ‘water’ (w) phase. Then, it can be surrounded by a spherical layer of immiscible fluid (‘oil’ phase (o)) suspended in another w-phase (w/o/w double emulsion), or suspended in the oil phase (w/o emulsion) [61]. W/o/w system is compatible with typical FACS fluidics and optics, so the analytical part can be conducted in a commercial FACS sorter. Analytics of droplets generated as w/o emulsion is typically run in laboratory-constructed microchips with electrodes embedded into the chip itself, due to incompatibility of the oily mobile phase with commercial FACS equipment. As pointed out earlier [57], using the droplet-based system reconstitutes a direct link between phenotype and genotype, which is lost in the case of secreted molecules and FACS-based sorting. In addition, the Authors demonstrated that using an intracellular concentration of a molecule (a measure compatible with FACS as a proxy of the total production rate of a secreted molecule is not adequate, making FADS specifically relevant for such an application.

The droplet generation and analytics have an incomparably higher capacity than any other high-throughput screening technique. With *Y. lipolytica*, the current technologies reach the droplets producing rate of ~ 10^6^/h, and sorting rate of over 10^5^ to 10^6^/h [5, 8, 35]. But the question is whether encapsulation in a droplet is compatible with the biology of the strictly aerobic, filamenting species?

The principal limitation of the actual ‘culturing’ of a living microbial cell in the pico-reactors is limited volume and mass transfer, which directly implies low provision of oxygen and nutrients, and, increasing with time, accumulation of metabolites. Furthermore, considering that FADS ensures a super-high resolution (at a single-cell level and not the average of a population), the incubation time of all the cells must be sufficient and comparable [14], which requires additional consideration in the continuous flow fluidics system. The other commonly pointed challenges are the investment costs, the need to couple the desired phenotype with some fluorescent product, the risk of the partitioning of hydrophobic products into the mobile phase (or the oil layer in w/o/w emulsions), and the low stability of the double w/o/w emulsions, which are compatible with FACS [6]. Moreover, the frequency of the droplet seeding at 1 cell/droplet is limited by the Poisson distribution [13], resulting in a high number of empty microdroplets. Some of these challenges were ameliorated by using fluorinated oil with good oxygen solubility [35] and fluorescent substrates of the analyzed enzymes [5], designing incubation chamber ensuring first-in-first-out dynamics of the droplets flow [14], pico-injection of the substrate to initially pre-selected droplets containing the desired 1 cell/ drop seeding rate [5]. Still, additional amendments were required to adapt the droplet-based microfluidic screening system to the use of *Y. lipolytica* [5] (discussed hereafter).

Another interesting ultra-high-throughput approach for yeast culturing was developed by [52, 53]. In that system, a multiplexed microfluidic chip was operated in a perfusion mode, so the cells were maintained in microliter volumes of a continuous stream of medium. In this system, the key limitations of closed pico-liter bioreactors (oxygen and nutrients availability, accumulation of metabolites) are no longer valid. In such a format, the screening system is scaled-down by 2000-fold, and the desired phenotypes can be identified in 2 to fourfold shorter times [52, 53]. Operation in a perfusion mode enabled precise control of the culturing conditions, making room for the process conditions optimization with this system. Notably, the results obtained with *K. phaffi* grown in such a microfluidic perfusion system were validated at larger scales, proving their effectiveness.

A similar system exploiting a 1 mL microfluidic bioreactor, operating in perfusion mode was also used with *Y. lipolytica* by [40]. More details on that system and ‘personalized’ adjustments required for *Y. lipolytica* are discussed hereafter (“*Y. lipolytica *in microfluidics” section).

## *Yarrowia lipolytica* ‘personalized’ scaling-down approaches

### MTP format

Considering the high interest in *Y. lipolytica* as a platform species in metabolic engineering and rProt production, urgent needs are placed on developing dedicated testing and screening platforms. However, as mentioned above, several characteristics of this yeast make the task very challenging. The principal limitations encountered in the high-throughput cultivations of *Y. lipolytica* and the state-of-the-art developments in this area are summarized below and in Table 2.

**Table 2:** Summary of the main limitations encountered in the high-throughput cultivations formats (O_96-well MTP and the pico-liter volume droplet) and solutions proposed in different studies

Limitations (including those specific to *Y. lipolytica*)	Proposed solutions with references
O_96-well MTP	
Insufficient aeration due to the use of plastic cover and ‘breathing freely film’	Stainless steel covers [48], sandwich covers [18]
Small working volumes for sampling	Multiplication at the cost of high-throughputness
Low buffering capacity, insufficient for longer cultivations	BioLector configured with an optional microfluidic module to be used to regulate pH (https://www.m2p-labs.com/bioreactors) Use of an optimized maleate-based buffering system [22]
Volume loss at the corners	Use of peripheral wells as cell-less humidifiers filled with medium or water at the cost of high-throughputness
Measurement limitations due to intensive growth (OD600 > 1.2), loss of linearity between the absorbance readout and cell density	Reading scattered light intensities rather than absorbance [1]
High surface tension/lack of turbulence	Flower-shaped plates from BioLector (https://www.m2p-labs.com/bioreactors) [1, 55] Square plates [17–20]
Position-on-the-plate-dependent oxygen diffusion rates to an individual well → differential profile of metabolites (difficulties in maintaining stable and comparable pH across the plate)	Use of individual air exchange systems above each well: holes above each well, but tightly sealing rants with a soft-silicone mat [17, 18] the independent vented cap above each well [60]
Pico-liter volume droplets	
Low oxygen availability (low nutrient availability and accumulation of metabolites were shown not to be limiting)	Use of fluorinated oil with good oxygen solubility [5, 35, 57] Increase the volume of oxygen-permeable fluorinated oil [35] Use of perfusion-mode microreactor (Air supply by gas diffusion through a sterile filter, online pH control) [40]
Poisson distribution of the droplet seeding—a high number of empty microdroplets	Adjustment of cell suspension density to match the desired seeding rate [5, 35] Removal of cellular aggregates prior to encapsulation by filtering through a 5 μm filter [35] Co-injection of the bioassay components and cells [35] Sub-sorting of the charged droplets from empty ones based on their size (use of a phenomenon of cell-containing droplet shrinkage [35]
Non-equal time of cell incubation prior to reading	For w/o droplet-type: designing incubation chamber ensuring first-in-first-out dynamics of the droplets flow and its incorporation into the microchip design [5, 14] For w/o/w droplet-time: additional incubation step [57]
Risk of the partitioning of hydrophobic products into the mobile phase (or the oil layer in w/o/w emulsions)	Use of synthetic reporters/alternative enzymatic substrates (fluorescent substrates) and its pico-injection to initially pre-selected droplets [5, 35]
Droplet integrity lost due to invasive growth	Use of a *Y. lipolytica* fil- strain (deleted for the Mhy1 gene, rendering the strain with a non-filamenting phenotype) (Hurtado and Rachubinski, 1999; [35])
Lipolytic activity-driven degradation of the oil phase	Use of fluorinated oil that is resistant to extracellular lipases due to fluorination of the aliphatic chains oil [5, 57]
Low stability of the emulsions	Addition of fluorosurfactant [5]
*Specific to w/o emulsion (requires laboratory-constructed microchips)*	
Investment costs for laboratory-constructed microchips with electrodes embedded into the chip itself	NA
Aggregation and adhesion of *Y. lipolytica* to hydrophobic surfaces	Proposed by [5]: Supplementation of the cell suspension with non-ionic detergent to limit the cells’ adhesion, Increase shear stress by adopting tubing with a smaller inner diameter (100 μm) to limit aggregation before encapsulation, Implementation of continuous stirring of the cell suspension during the encapsulation to prevent cell sedimentation, Adjustment of the initial cell density to approach the desired seeding rate of 0.03–0.1 cells/droplet [35] further decreased the inner diameter of the tubing to 50 μm

In a series of works [1, 5, 35, 38, 39] a French group transformed *Y. lipolytica* high-throughput screens from typical 96-well MTPs to a versatile and ultra-high-throughput FADS system.

**Fig. 5:**
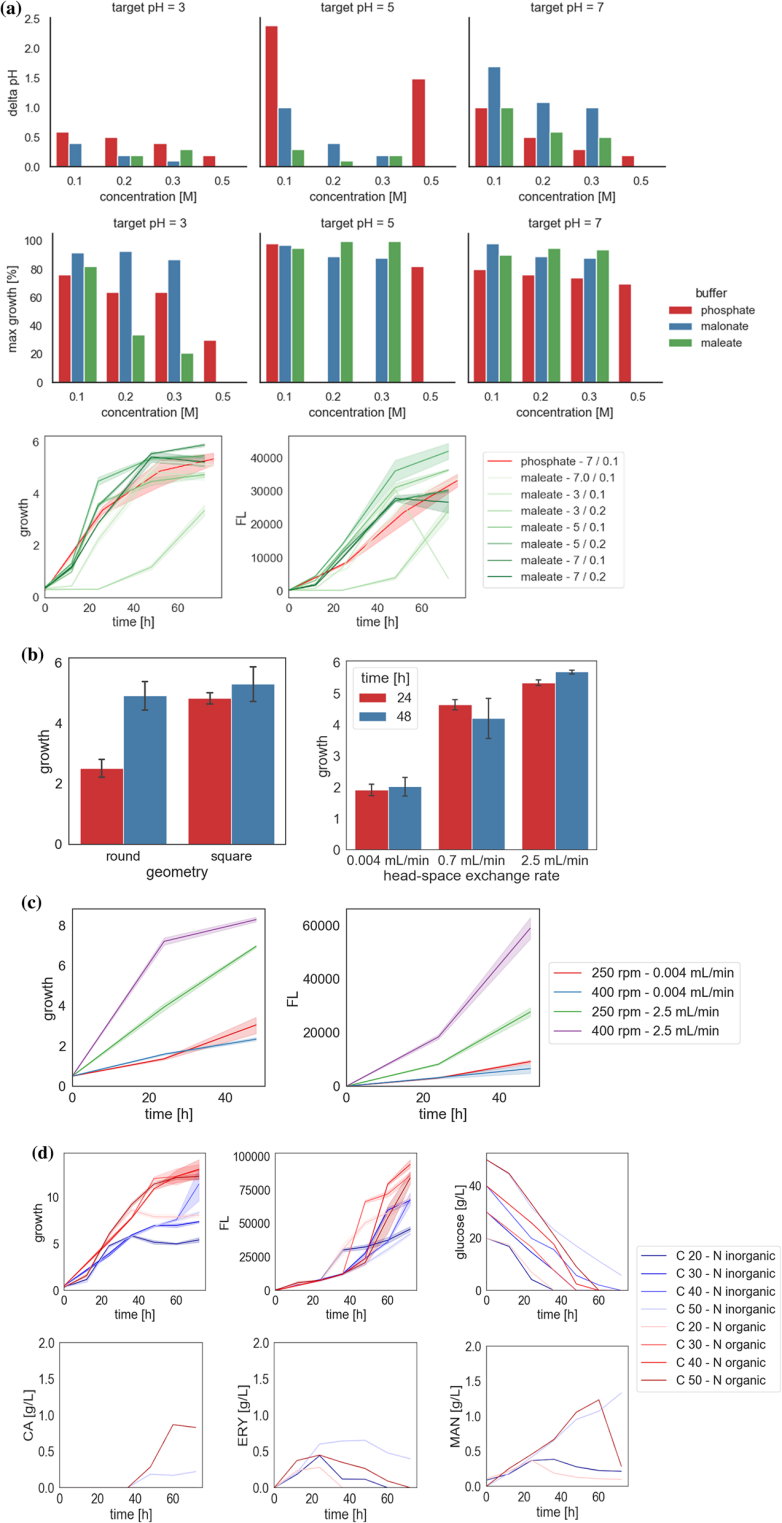
Optimization of a protocol for high-throughput *Y. lipolytica* cultivation in square-24-well-MTPs. Selected data from [22] are graphically presented here: (**A**) buffering capacity of selected buffers and growth of Y. lipolytica cells in the specifically-buffered 3xYNB medium, (**B**) growth of Y. lipolytica in O- and □-shaped 24-well MTPs, and under different ‘sandwich covers’ of specified air-exchange volume; (**C**) growth of Y. lipolytica in □-shaped 24-well MTPs under different ‘sandwich covers’ and at different shaking frequencies; (**D**) optimization of carbon load (C20, C30, C40, and C50) at fixed nitrogen load of 15 g/L (organic—casein hydrolysate; inorganic—ammonium sulfate) based on results on growth, fluorescence, and concentration of the main metabolites (CA, ERY, MAN)

In their initial work, [38] used the typical O-96-well MTPs filled in two-thirds (200 per 300 μL in total) of culture medium buffered with 50 mM phosphate buffer at pH 6.8. To address the problem of evaporation (illustrated in Fig. 4) the peripheral wells of the plate were filled with water. Cultures were conducted in a plate reader Biotek Synergy MX (Biotek Instruments) for 48 h at 28 °C with continuous agitation. In that work, the MTP culturing was used to manage a large library of strains developed from high-throughput transformation protocol, and not much focus was placed on its optimization, and no assessment of the well-to-well variation was provided. A similar high-throughput cultivation protocol was adopted in the following screens, using the same plate format and the reader [39, 54]. [39] noted that the cultures could be continued only up to 24 h, as later the OD600 readouts were > 1.2, above which the correlation between OD and cell density is not linear anymore. We found that point relevant in our experiments with *Y. lipolytica*. In addition, the necessity of shortening the culturing time in the automated plate reader used by the Authors was appropriate due to the limited buffering capacity of 50 mM phosphate buffer, which was probably insufficient for longer cultivations [22]. In the following work, [1, 55] used a specialized MTP shaker-incubator-reader, BioLector (mp2-labs, Baesweiler, Germany), and unique MTPs—48-well Flower-Plates (mp2-labs). The topology of these plates is highly compatible with the strictly aerobic *Y. lipolytica*. Each inner edge of the ‘six-petal flower’ acts as a baffle, enhancing the oxygen transfer rate substantially (up to > 0.11 mol/L/h). *Y. lipolytica* strains were cultured in either rich or minimal medium buffered with phosphate buffer at pH 6.8, under 28 °C and constant agitation (1200 rpm, shaking diameter = 3 mm, orbital) in a working volume of 800 µL (possible filling volume up to 2400 μL). In the BioLector system, growth (and fluorescence) can be continuously monitored by measuring scattered light intensities, which offers a huge advantage over the formerly used OD600, especially with high-cell-density-growing *Y. lipolytica*. As evidenced in that work, scattered light measurements in *Y. lipolytica* cultures displayed a linear correlation with those of OD600nm up to 115 units. The assessment of dry cellular weight concentration using OD600 as a proxy could reach maximally 58 g/L, while using scattered light—as much as 100 g/L. In [1], the cultures were continued for > 100 h, and pH was stabilized at pH 6.8 with 50 mM phosphate buffer. Our previous analyses [22] (Fig. 5A) showed that even twice stronger phosphate buffer (100 mM) does not possess sufficient buffering capacity to stably maintain pH at ~ 7.0 for 48 h in *Y. lipolytica* cultures (drop to pH 6.0 after 48 h; noteworthy—a different culturing medium was used). Currently, the BioLector system can be configured with an optional microfluidic module, which may be used to regulate pH (2 feeding lines are available). Such functionality is highly relevant for *Y. lipolytica* cultures, which tend to strongly acidify the medium upon growth.

High throughput screening in 48-well plates of an extensive library of *Y. lipolytica* strain was conducted by [60]. The screening was performed in MicroScreen system (Gering Ltd., China) in 1 mL of mineral medium (per 3 mL total volume), with an initial pH of 6.0 (no buffer provided) for up to 36 h. According to the system’s provider, the device can ensure shaking up to 1000 rpm (for *Y. lipolytica* the Authors used 800 rpm), but foremost, the plates are equipped with the independent vented cap above each well, which altogether contributes to higher k_L_a levels. The important drawback of that system is that in growth tracking it uses OD600 measurements with the upper limit of 5.0. Still, it allows to calculate the initial growth rate, which is sufficient to assess the strain’s robustness.

Knowing the limitations of *Y. lipolytica* culturing and its high oxygen demands, [48] tested different covering systems in combination with 96-deep-well plates for *Y. lipolytica* screens. The experiment aimed at screening over 1000 *Y. lipolytica* clones in a high-glucose medium (900 μL working volume in 2 mL total well volume) with shaking at 220 rpm for 10 days. Inoculated deep-well plates were either sealed with ‘breathing freely film’ or covered with paper and stainless steel covers. When stainless steel covers were used, the growth of *Y. lipolytica* strains was 10- to 20-fold higher, when compared to the cultures sealed with the film, and by only 60% worse than when grown in shake flasks (using the same carbon source load). Control and regulation of pH were not addressed in that research.

### *Y. lipolytica* in microfluidics

The very first implementation of microfluidics technology to *Y. lipolytica* high-throughput screens was reported by [5]. To screen for strains secreting hydrolases, the Authors developed two microfluidics devices: (i) a dropmaker and (ii) an integrated screening device. The former encapsulated *Y. lipolytica* cells into 20 picoliter droplets at nearly 10^7^ /h rate, creating a w/o-type emulsion in fluorinated oil. The mobile phase was enriched in fluorosurfactant to stabilize the emulsion. Noteworthy, the selected oil phase was: (i) resistant to extracellular lipases due to fluorination of the aliphatic chains oil [12, 56], and (ii) permeable to oxygen–both characteristics of key importance considering *Y. lipolytica*’s biology. The Authors reliably assessed the limitations of performing *Y. lipolytica* screens in the microfluidics system, and introduced the necessary ameliorations. For example, it was noted that the yeast cells display affinity to hydrophobic substrates, and a tendency to aggregate on the channel walls. To overcome these problems, the Authors implemented several specific amendments: (i) supplemented the cell suspension with non-ionic detergent to limit the cells’ adhesion, (ii) increased shear stress by adopting tubing with a smaller inner diameter (100 μm) to limit aggregation before encapsulation. In addition, the system’s operation was improved by (iii) the implementation of continuous stirring of the cell suspension during the encapsulation to prevent cell sedimentation, and (iv) adjustment of the initial cell density to approach the desired seeding rate of 0.03–0.1 cells/droplet [5]. Following the droplet formation, the seeded pico-bioreactors were set in operation for 16 h at 28 °C. Over that period, the cells divided and produced the targeted enzymes. The demonstration that *Y. lipolytica* cells could actually replicate inside the droplets is of key importance, proving that the system can be truly regarded as ‘cultivation in pico-bioreactors’ and not just ‘coating and incubation’. It was the first reliable system accompanied by a detailed protocol for managing the pico-cultivation of *Y. lipolytica* for high-throughput screens.

[57] developed a double emulsion w/o/w FADS system to screen for *Y. lipolytica* strains producing and secreting high amounts of riboflavin, and compared such a screening technology with the typical single-cell FACS sorting. In both cases, a two-step cascade of sequential FA(C/D)S enrichment was used to isolate the final ‘hyper-producer’ populations. The aim was to evaluate if successive enrichment and sorting using either of the two methods would impact the outcome–performance of the best producer. Technically, the double emulsion was obtained by recurring flow-focusing-driven encapsulation: (i) cell suspension in fluorinated oil with amphiphilic block copolymer surfactant, followed by the droplets collection and incubation for 3 days to allow for riboflavin production inside the pico-bioreactors; (ii) entrapment of the w/o emulsion from step (i) in the outer phase water solution. To enrich the initial population in the best producers, two subsequent FACS sorts with increasing stringency were applied. Intermittent incubations (3 days, 30 °C) between the sorts allowed to spontaneously phase separate. It was not shown if *Y. lipolytica* propagated inside the droplets over the 3 days between w/o and w/o/w emulsion generation. The period of 3 days is fourfold higher than the 18 h adopted by [5]. However, the cells could produce the target molecule, when incubated in the w/o emulsion, and be screened for its concentration, meaning that the metabolism was active. The developed FADS technology proved to be significantly more efficient in selecting *Y. lipolytica* producing higher amounts of riboflavin in total, considering the intra- and extracellular levels.

Most recent research implementing microfluidics to *Y. lipolytica* aimed at screening large libraries of strains secreting antibodies in their final, water-soluble format [35]. The Authors developed and optimized a protocol for growing and producing full-length antibodies by *Y. lipolytica* strains in pico-bioreactors, and applied a microfluidics approach to sort and recover target-specific antibody-secreting yeasts. On their way, several interesting aspects were addressed. Foremost, the host strain was deleted for the *Mhy1* gene, rendering the strain with a non-filamenting phenotype, which is highly relevant for any microfluidics approach and important for droplet integrity. The chip channel diameter was further decreased to 50 μm (when compared to the solution proposed by [5]), to prevent *Y. lipolytica* adhesion to the walls. The 30 picoliter volume droplets were formed in w/o emulsion, using the hydrofluoroether oil phase. The cellular aggregates were removed prior to encapsulation by filtering through a 5 μm filter. Yeast cell suspension density was adjusted to match the desired seeding rate. Charging the drops with the yeast cells and the bioassay components was conducted by co-injection, followed by overnight growth of the cells and production of the antibodies. As concluded by the Authors, growth and product synthesis in pico-bioreactors were not limited by nutrient availability and metabolite accumulation. The only limiting factor was oxygen availability. To cope with this issue, the volume of oxygen-permeable fluorinated oil was increased, which, as stated, directly increased the oxygen provision. At the sorting stage, the Authors took advantage of the commonly occurring phenomenon of cell-containing droplet shrinkage. In this way, the seeded droplets were sorted from empty ones based on size, without the need for an extra cell tracer. Following the microchip sorting, the droplets containing the target cells were deflected by dielectrophoresis to recover the secreting yeasts. Due to high survival over the sorting process and rapid out-growth after the procedure, *Y. lipolytica* strains could be reused in further screenings—for enrichment studies or selection according to different criteria.

As briefly mentioned above, [40] reported on the use of a 1 mL microfluidic perfusion bioreactor to study fundamental aspects of *Y. lipolytica* biology. Technically, the cultures were conducted in a commercial single-use 1 mL microbioreactor chip (Pharyx Inc., USA). The microreactor was equipped with optical density, oxygen, pH, and temperature probes to ensure precise control of the process parameters. Air was supplied by gas diffusion through a sterile filter, pH was controlled by the automated addition of strongly diluted NaHCO_3_ (1 mM), while a temperature of 28 °C was by regulated a heating element located at the bottom of the device. The growth chamber comprised three interconnected 500 μL sections, out of which two were occupied to secure both the 1 mL working volume and the mixing. More details on the system can be found in [7, 36, 37]. After several volume exchanges, *Y. lipolytica* culture reached a steady-state highlighted by a stable biomass concentration (~ 5 g of dry cellular weight/L), stable oxygen saturation (above 40%), and, most importantly for that experiment, a stable residual glucose concentration < 1 mg/L. The latter was of key importance, as the Authors investigated the relationship between residual glucose levels and dimorphism in *Y. lipolytica*. Notably, *Y. lipolytica* displayed high viability in such a system (above 97%). By definition, exploring dimorphism in a microfluidics system is a challenging task, due to the high risk of clogging the tubings by filaments. Being aware of this, the Authors first simulated conditions promoting ovoid morphotype (then, *Y. lipolytica* cell is ~ 4.4 μm in diameter). Further ‘personalization’ of the experiment consisted of adjusting the time of the ‘filamentation-inducing state’, disallowing full development of the long filaments. Using such a system it was possible to prove that the dimorphic shift is dependent on the residual glucose levels. Very precise determination of the threshold level (0.35−0.37 mg/L) was possible due to the incomparably high resolution of the perfusion microbioreactor, and the excellent technical execution of the experiment. In that experiment, the microfluidics system was not used for different phenotype comparisons, so it was also not assessed for its screening capacity, run-to-run variability, and throughputness. However, the observations done with the microfluidics were validated in a standard 1 L bioreactor, which confirmed the operability of the system and its usefulness for the dimorphic *Y. lipolytica*.

## Protocol for high-throughput screens of *Y. lipolytica* strains in square-MTPs

Our recent project required the development of a high-throughput screening protocol to reliably compare phenotypes of *Y. lipolytica* strains, individually over-expressing a single transcription factor per strain [22, 25, 39]. Knowing the key role of transcription factors in modulating cellular biology, we expected that the spectrum of obtained phenotypes (for 125 strains tested) would be very wide. Hence, developing a single protocol, that matches the requirements of all the strains would be a significant challenge. The first trials conducted in the standard O_96-well plates turned out to be unsuccessful due to low repeatability. Even if the strains grew and the reporter rProt was detected, the repeatability between successive runs was low and implementation of different environmental variables did not allow to determine any statistically significant factors affecting the observed outcomes. Considering the number of strains and conditions to be tested (rendering altogether 26,000 cultures), and limited budget (disallowing purchase of BioLector and consumables), we set for the development of a culturing protocol that would enable full development of a variables-driven phenotype, without uncontrolled infliction of unknown environmental factors [22].

From an array of different MTPs available on the market, reusable □_24-well MTPs (www.enzyscreen.com) were chosen. Reusability, and hence limitation of plastic waste, was a huge asset of the system. The Duetz system is well-developed and supported by reliable studies [17–20]. The usefulness of that system was proved for culturing different bacteria species [18–20], filamentous fungi [32], the model yeast species *S. cerevisiae* [28, 31], and recently also–*Y. lipolytica* [27, 30, 47, 49]. In that project, a system enabling a controlled variable-driven phenotype development was of key importance. Therefore, several key parameters, like pH maintenance, oxygen availability, and sufficient nutrient provision throughout the culturing time were carefully investigated.

Since that research relied on batch cultivations, and the number of cultures run in parallel disallowed manual correction of the cultures’ acidity in response to the cells’ metabolism, it was necessary to establish a robust buffering system [22]. That experimental plan covered a (problematic) pH range spanning pH 3–5–7. The aim was to have a buffer that enables buffering at the three levels using the same chemical compound,with the final change in the set pH value no higher than 0.5 pH unit. Considering the metabolic characteristics of *Y. lipolytica*, buffers based on citric acid and acetic acid were immediately eliminated, as the components would be consumed [3]. Hence, the investigated set covered: phosphate, malonate, maleate, MES, and carbonate buffers at concentrations from 0.1 to 0.5 M (Fig. 5A; based on data presented in [22]). Phosphate buffer was a good buffering system at 0.3 M concentration but only close to its pKa—pH 3.0 and 7.0 (concentration of the buffer’s acid and conjugate base forms are equal). Even at a concentration of 0.5 M, it had no buffering capacity close to pH 5.0. In addition, 0.5 M phosphate buffer at pH 3 strongly limited the growth of *Y. lipolytica* (30% of the maximum read for all the conditions considered; Fig. 5A). Carbonate and MES were considered complementary buffers to the phosphate buffer at pH 5.0. However, at none of the concentrations tested (0.1 and 0.5 M), they secured sufficient buffering capacity for *Y. lipolytica* metabolism–the drop in pH was by more than 1.5 pH unit (from pH 5.0 to pH 3.5 to pH 2.3). The final two tested buffers were based on dicarboxylic acids having two pKa values. The pKa of maleic acid is 1.9 and 6.2, while of malonic acid—2.83 and 5.69 (PubChem data). Since these compounds were not previously used for buffering *Y. lipolytica* cultures, possible consumption of the two chemical compounds by *Y. lipolytica* was tested. The change in their concentration at the end of culturing (HPLC; data not showed) was within a technical error, indicating that *Y. lipolytica* does not utilize the compounds and they can be used as buffering agents.

Considering the target pH levels (3, 5, 7), and that the buffering capacity of a buffer is highest when the pH is within one unit of the pKa value, the maleate buffer would be more adequate to buffer at pH 7, while malonate–at pH 3 and 5. Indeed, malonate at a concentration of > 0.2 M buffered *Y. lipolytica* cultures very well at acidic pH, but turned out to lack the capacity at neutral pH. On the other hand, 0.2 M maleic buffer was very efficient in buffering pH at 5 and 7. At pH 3 its buffering capacity was also sufficient, but it limited *Y. lipolytica* growth when its concentration was > 0.2 M (to 20–30% of the maximum; Fig. 5A). Hence, to meet the requirement of having a single chemical compound in all the buffers, and considering that the buffering capacity of 0.1 M maleate was sufficient at pH 3, an optimal buffering system for *Y. lipolytica* cultures comprised of maleate buffer at 0.1 M for pH 3.0, and at 0.2 M for pH 5.0 and 7.0. The potential toxicity of the maleic buffer was investigated in comparison to phosphate buffer (both pH 7.0, 0.1 M); demonstrating a lack of the limiting effect (Fig. 5A). To ensure that no uncontrolled variable is introduced by using the buffer at different concentrations, osmolality in the medium formulation was experimentally determined. The 0.1 M vs 0.2 M maleate buffer yielded osmolality of 1.27- to 1.78-fold higher than the standard YPG medium [22], which is away from the stress level (nearly fivefold higher [34]). Growth and rProt synthesis by *Y. lipolytica* in media buffered with the developed buffering system was studied, proving its functionality (Fig. 5A). Using such a buffering system, it was established that *Y. lipolytica* grows and produces rProt most efficiently at pH 5.0. Initial growth was significantly slower at pH 3, but finally reached only slightly lower yields at the stationary phase (8% lower biomass than at pH 5). On the other hand, rProt synthesis at the acidic pH was significantly diminished (by nearly 40% vs pH 5). This observation well aligned with previous results conducted in bioreactors with a different reporter rProt, showing that a pH of 3 is a limiting factor of rProt synthesis, especially when combined with some other stress factor [23]. No such differences were observed between growth and rProt synthesis by *Y. lipolytica* in pH 5 and 7.

Another parameter investigated and optimized in that research was oxygen level supply [22]. The aim was to orchestrate the type of consumables, equipment, shaking frequency, and culture volume to generate two distinctly different conditions – ‘high’ and ‘low’ oxygen supply. It was noted that when *Y. lipolytica* is cultured in □_24-well vs O_24-well MTPs, according to the manufacturer’s suggestions (2.5 mL in the former, 1 mL in the latter), biomass in the stationary phase is not much different (by 7% lower in the O-shaped MTPs; please compare also Fig. 1 and Table 1), but the stationary phase is reached by nearly 24 h faster in the square-shaped MTPs (Fig. 5B–based on data presented in [22]). Considering the scope of that investigation (a strain library screening under multiple conditions), such a shift in time was of high interest. To simulate conditions of clearly divergent oxygen supply, covers with different nominal head-space exchange rates were tested (‘sandwich covers’ of 0.004, 0.7, and 2.5 mL/min). Technically, the differences were implemented by differing the type of the silicone mat and the diameter of the openings above each well. According to [18] an average aerobic microbial culture needs an air supply of one working volume per minute (1 vvm; in our case—2.5 mL). If supplied, the oxygen concentration in the headspace is maintained at > 18% (v/v), even at very high oxygen consumption rates (40 mmol oxygen/L/minute)*.* Depending on the cover type, the growth of *Y. lipolytica* nearly linearly decreased along with a decrease in the nominal head-space exchange rates (r = 0.84 across all the pH values; r = 0.91 for pH 5); at 0.004 mL/min reaching only 36% of its value achieved at 2.5 mL/min (Fig. 5B). A comparable decrease (to 33%) was read for the rProt synthesis level expressed in biomass-normalized values, indicating a direct effect of oxygen provision on rProt synthesis. This experiment showed that the ‘sandwich cover’ system solution offered by (www.enzyscreen.com) works efficiently in simulating different aeration rates in *Y. lipolytica* cultures. Moreover, the difference in oxygen supply driven by the use of 0.07 and 2.5 mL/min sandwich covers was not sufficient, to use them to simulate the distinctly different aeration conditions; hence, the middle variant of 0.07 mL/min was eliminated from further studies.

Selection of the ‘sandwich cover’ type was done at the same shaking parameters (shaking amplitude of 1.91 cm, and frequency of 250 rpm). To investigate if changes in shaking frequencies would further modulate *Y. lipolytica* growth under the selected ‘sandwich covers’, shaking frequencies up to 500 rpm were tested (amplitude remained constant) (Fig. 5C). Such an experiment would also answer the question of whether the use of 2.5 mL/min sandwich covers suffice the oxygen demand by *Y. lipolytica* at the used shaking frequency. In that experiment, the risk of cross-well contamination was monitored by a checkered inoculation pattern. As observed, shaking at 500 rpm leads to splashing of the culture on the cover imposing a risk of cross-contamination, so this variant was eliminated. Shaking at a frequency of 400 rpm led to a significantly higher rate of growth and rProt synthesis vs shaking at 250 rpm, but only when 2.5 mL/min sandwich covers were used (no such an effect was observed under the covers 0.004 mL/min). The observed increase in those parameters suggests that the air supply of 1 vvm at 250 rpm is not sufficient for *Y. lipolytica*’s robust metabolism. Such a result is concurrent with previous observations done with *Y. lipolytica* bioreactor cultures, where at least 2 vvm of air had to be supplied to meet the desired 20% oxygen concentration (e.g. [33]). Notably, when shaking at 400 rpm, the level of growth and rProt amount in 24 h of culture was the same, as in 48 h of culturing when shaking at 250 rpm. It is a substantial increase in pace, greatly increasing the throughputness of the system. No effect of shaking frequency on the amounts of biomass and rProts under air-exchange-limiting covers use, indicates the efficiency of the system in securing a controlled head-space exchange rate.

The supply of carbon and nitrogen sources was also subjected to adjustment, but in combination. The aim was to secure sufficient amounts of carbon and nitrogen until the end of culturing, without the risk of introducing a starvation period (which would be an additional, uncontrolled variable affecting the phenotypes). That experiment was conducted with the fastest-growing strain from the library [39] to determine the minimum sufficient amounts of carbon (20 to 50 g/L) and nitrogen (5 to 15 g/L, organic and inorganic, Fig. 5D). Under the adopted culturing conditions, nitrogen load at 5 g/L was insufficient, leading to limited biomass growth and carbon source consumption (by 34% when inorganic nitrogen was used; partly compensated when organic nitrogen was supplied; as shown previously [10]). Using nitrogen at 15 g/L and carbon at > 30 g/L, eliminated the risk of a starvation period occurrence, as by the end of the culture, still some minimal level of the nutrient was present. Profile of CA, ERY, and MAN concentration support that thesis, as within the investigated time (up to 48 h), these compounds were not reconsumed in those culture variants (Fig. 5D).

Further notions from the high-throughput cultivation protocol optimization, more related to the research question than to the technical aspects of the cultivation, are presented in the accompanying article [22] and the open, freely accessible database YaliFunTome (https://sparrow.up.poznan.pl/tsdatabase/) presenting the results obtained with the protocol.

## Summary and conclusions

Literature review and our own experience show that high-throughput cultivation protocols require extra consideration when being adjusted to *Y. lipolytica* characteristics. The vast knowledge accumulated on its biology is of great assistance in rationally designing the amendments. Technological progress offers many solutions that adequately respond to challenges from the yeast species biology. The authors of the original research covered by this review provided evidence for the repeatability, high capacity, and operability of their systems for *Y. lipolytica* cultivations. Using such dedicated and validated protocols may greatly aid the cumbersome screening stage of a synthetic biology-based clone library or run parallelized cultures for extensive experimental design.

### Supplementary Information

Additional file 1.

## Data Availability

The datasets generated for the ‘proof-of-concept’ experiment are available from Mendeley Data [11]. YaliFunTome database, accompanying “Protocol for high-throughput screens of *Y. lipolytica *strains in square-MTPs” section. is publicly available free of charge at: https://sparrow.up.poznan.pl/tsdatabase/

## References

[1] Back A, Rossignol T, Krier F, Nicaud JM, Dhulster P (2016). High-throughput fermentation screening for the yeast *Yarrowia lipolytica* with real-time monitoring of biomass and lipid production. Microb Cell Fact.

[2] Baret J-C, Miller OJ, Taly V, Ryckelynck M, El-Harrak A, Frenz L, Rick C, Samuels ML, Hutchison JB, Agresti JJ, Link DR, Weitz DA, Griffiths AD (2009). Fluorescence-activated droplet sorting (FADS): efficient microfluidic cell sorting based on enzymatic activity. Lab Chip.

[3] Barth G, Gaillardin C. *Yarrowia lipolytica*, In: Wolf K. Nonconventional yeasts in biotechnology a handbook. Springer Berlin Heidelberg. 1996, pp. 313–388. 10.1007/978-3-642-79856-6

[4] Baryshnikova A, Costanzo M, Kim Y, Ding H, Koh J, Toufighi K, Youn JY, Ou J, San Luis BJ, Bandyopadhyay S, Hibbs M, Hess D, Gingras AC, Bader GD, Troyanskaya OG, Brown GW, Andrews B, Boone C, Myers CL (2010). Quantitative analysis of fitness and genetic interactions in yeast on a genome scale. Nat Methods.

[5] Beneyton T, Thomas S, Griffiths AD, Nicaud JM, Drevelle A, Rossignol T (2017). Droplet-based microfluidic high-throughput screening of heterologous enzymes secreted by the yeast *Yarrowia lipolytica*. Microb Cell Fact.

[6] Boitard L, Cottinet D, Kleinschmitt C, Bremond N, Baudry J, Yvert G, Bibette J (2012). Monitoring single-cell bioenergetics via the coarsening of emulsion droplets. Proc Natl Acad Sci.

[7] Bower DM, Lee KS, Ram RJ, Prather KLJ (2012). Fed-batch microbioreactor platform for scale down and analysis of a plasmid DNA production process. Biotechnol Bioeng.

[8] Bowman EK, Alper HS (2020). Microdroplet-assisted screening of biomolecule production for metabolic engineering applications. Trends Biotechnol.

[9] Celińska E, Białas W, Borkowska M, Grajek W (2015). Cloning, expression, and purification of insect (*Sitophilus oryzae*) alpha-amylase, able to digest granular starch, in *Yarrowia lipolytica* host. Appl Microbiol Biotechnol.

[10] Celińska E, Borkowska M, Białas W (2017). Enhanced production of insect raw-starch-digesting alpha-amylase accompanied by high erythritol synthesis in recombinant *Yarrowia lipolytica* fed-batch cultures at high-cell-densities. Process Biochem.

[11] Celińska E, Gorczyca M (2024). Comparison of *Yarrowia lipolytica* growth and rProt synthesis when cultured in different vessels. Mendeley Data.

[12] Chowdhury MS, Zheng W, Kumari S, Heyman J, Zhang X, Dey P, Weitz DA, Haag R (2019). Dendronized fluorosurfactant for highly stable water-in-fluorinated oil emulsions with minimal inter-droplet transfer of small molecules. Nat Commun.

[13] Clausell-Tormos J, Lieber D, Baret J-C, El-Harrak A, Miller OJ, Frenz L, Blouwolff J, Humphry KJ, Köster S, Duan H, Holtze C, Weitz DA, Griffiths AD, Merten CA (2008). Droplet-based microfluidic platforms for the encapsulation and screening of mammalian cells and multicellular organisms. Chem Biol.

[14] Dai J, Kim HS, Guzman AR, Shim W-B, Han A (2016). A large-scale on-chip droplet incubation chamber enables equal microbial culture time. RSC Adv.

[15] Deere D, Shen J, Vesey G, Bell P, Bissinger P, Veal D (1998). Flow cytometry and cell sorting for yeast viability assessment and cell selection. Yeast.

[16] Doig SD, Pickering SCR, Lye GJ, Baganz F (2005). Modelling surface aeration rates in shaken microtitre plates using dimensionless groups. Chem Eng Sci.

[17] Duetz WA (2007). Microtiter plates as mini-bioreactors: miniaturization of fermentation methods. Trends Microbiol.

[18] Duetz WA, Rüedi L, Hermann R, O’Connor K, Büchs J, Witholt B (2000). Methods for intense aeration, growth, storage, and replication of bacterial strains in microtiter plates. Appl Environ Microbiol.

[19] Duetz WA, Witholt B (2004). Oxygen transfer by orbital shaking of square vessels and deepwell microtiter plates of various dimensions. Biochem Eng J.

[20] Duetz WA, Witholt B (2001). Effectiveness of orbital shaking for the aeration of suspended bacterial cultures in square-deepwell microtiter plates. Biochem Eng J.

[21] Ferreira-Torres C, Micheletti M, Lye GJ (2005). Microscale process evaluation of recombinant biocatalyst libraries: application to Baeyer-Villiger monooxygenase catalysed lactone synthesis. Bioprocess Biosyst Eng.

[22] Gorczyca M, Białas W, Nicaud J-M, Celińska E (2024). ‘Mother(Nature) knows best’–hijacking nature-designed transcriptional programs for enhancing stress resistance and protein production in *Yarrowia lipolytica*; presentation of YaliFunTome database. Microb Cell Fact.

[23] Gorczyca M, Każmierczak J, Fickers P, Celińska E (2022). Synthesis of secretory proteins in *Yarrowia lipolytica*: effect of combined stress factors and metabolic load. Int J Mol Sci.

[24] Gorczyca M, Kaźmierczak J, Steels S, Fickers P, Celińska E (2020). Impact of oxygen availability on heterologous geneexpression and polypeptide secretion dynamics in *Yarrowia lipolytica*-based protein production platforms. Yeast.

[25] Gorczyca M, Nicaud J-M, Celińska E (2023). Transcription factors enhancing synthesis of recombinant proteins and resistance to stress in *Yarrowia lipolytica*. Appl Microbiol Biotechnol.

[26] Hermann R, Lehmann M, Büchs J (2003). Characterization of gas-liquid mass transfer phenomena in microtiter plates. Biotechnol Bioeng.

[27] Holkenbrink C, Dam MI, Kildegaard KR, Beder J, Dahlin J, Domenech Belda D, Borodina I (2018). EasyCloneYALI: CRISPR/Cas9-based synthetic toolbox for engineering of the yeast *Yarrowia lipolytica*. Biotechnol J.

[28] Jessop-Fabre MM, Jakočiūnas T, Stovicek V, Dai Z, Jensen MK, Keasling JD, Borodina I (2016). EasyClone-MarkerFree: a vector toolkit for marker-less integration of genes into Saccharomyces cerevisiae via CRISPR-Cas9. Biotechnol J.

[29] Kensy F, Zimmermann HF, Knabben I, Anderlei T, Trauthwein H, Dingerdissen U, Büchs J (2005). Oxygen transfer phenomena in 48-well microtiter plates: determination by optical monitoring of sulfite oxidation and verification by real-time measurement during microbial growth. Biotechnol Bioeng.

[30] Kildegaard KR, Adiego-Pérez B, Doménech Belda D, Khangura JK, Holkenbrink C, Borodina I (2017). Engineering of *Yarrowia lipolytica* for production of astaxanthin. Synth Syst Biotechnol.

[31] Kildegaard KR, Tramontin LRR, Chekina K, Li M, Goedecke TJ, Kristensen M, Borodina I (2019). CRISPR/Cas9-RNA interference system for combinatorial metabolic engineering of *Saccharomyces cerevisiae*. Yeast.

[32] Kosa G, Vuoristo KS, Horn SJ, Zimmermann B, Afseth NK, Kohler A, Shapaval V (2018). Assessment of the scalability of a microtiter plate system for screening of oleaginous microorganisms. Appl Microbiol Biotechnol.

[33] Kubiak M, Borkowska M, Białas W, Korpys P, Celińska E (2019). Feeding strategy impacts heterologous protein production in *Yarrowia lipolytica* fed-batch cultures—insight into the role of osmolarity. Yeast.

[34] Kubiak-Szymendera M, Skupien-Rabian B, Jankowska U, Celińska E (2022). Hyperosmolarity adversely impacts recombinant protein synthesis by *Yarrowia lipolytica*—molecular background revealed by quantitative proteomics. Appl Microbiol Biotechnol.

[35] Lebrun E, Shenshin V, Plaire C, Vigneres V, Pizette T, Dumas B, Nicaud JM, Mottet G (2023). Efficient full-length IgG secretion and sorting from single yeast clones in droplet picoreactors. Lab Chip.

[36] Lee KS, Boccazzi P, Sinskey AJ, Ram RJ (2011). Microfluidic chemostat and turbidostat with flow rate, oxygen, and temperature control for dynamic continuous culture. Lab Chip.

[37] Lee KS, Ram RJ (2009). Plastic–PDMS bonding for high pressure hydrolytically stable active microfluidics. Lab Chip.

[38] Leplat C, Nicaud JM, Rossignol T (2015). High-throughput transformation method for *Yarrowia lipolytica* mutant library screening. FEMS Yeast Res.

[39] Leplat C, Nicaud J-MM, Rossignol T (2018). Overexpression screen reveals transcription factors involved in lipid accumulation in *Yarrowia lipolytica*. FEMS Yeast Res.

[40] Lesage J, Timoumi A, Cenard S, Lombard E, Lee HLT, Guillouet SE, Gorret N (2021). Accelerostat study in conventional and microfluidic bioreactors to assess the key role of residual glucose in the dimorphic transition of *Yarrowia lipolytica* in response to environmental stimuli. N Biotechnol.

[41] Li Z, Vizeacoumar FJ, Bahr S, Li J, Warringer J, Vizeacoumar FS, Min R, VanderSluis B, Bellay J, DeVit M, Fleming JA, Stephens A, Haase J, Lin Z-Y, Baryshnikova A, Lu H, Yan Z, Jin K, Barker S, Datti A, Giaever G, Nislow C, Bulawa C, Myers CL, Costanzo M, Gingras A-C, Zhang Z, Blomberg A, Bloom K, Andrews B, Boone C (2011). Systematic exploration of essential yeast gene function with temperature-sensitive mutants. Nat Biotechnol.

[42] Liccioli T, Tran TMT, Cozzolino D, Jiranek V, Chambers PJ, Schmidt SA (2011). Microvinification—how small can we go?. Appl Microbiol Biotechnol.

[43] Maier U, Büchs J.Gas-Flüssigkeits-Stofftransfer im Schüttelkolben. Aachen, Techn. Hochsch. 2002.

[44] Maier U, Losen M, Büchs J. Advances in understanding and modeling the gas-liquid mass transfer in shake flasks, In: Biochemical Engineering Journal. Elsevier. 2004, pp. 155–167. 10.1016/S1369-703X(03)00174-8

[45] Marešová L, Sychrová H (2007). Applications of a microplate reader in yeast physiology research. Biotechniques.

[46] Meier K, Klöckner W, Bonhage B, Antonov E, Regestein L, Büchs J (2016). Correlation for the maximum oxygen transfer capacity in shake flasks for a wide range of operating conditions and for different culture media. Biochem Eng J.

[47] Milne N, Tramontin LRR, Borodina I (2020). A teaching protocol demonstrating the use of EasyClone and CRISPR/Cas9 for metabolic engineering of *Saccharomyces cerevisiae* and *Yarrowia lipolytica*. FEMS Yeast Res.

[48] Qiu X, Xu P, Zhao X, Du G, Zhang J, Li J (2020). Combining genetically-encoded biosensors with high throughput strain screening to maximize erythritol production in *Yarrowia lipolytica*. Metab Eng.

[49] Sáez-Sáez J, Wang G, Marella ER, Sudarsan S, Cernuda Pastor M, Borodina I (2020). Engineering the oleaginous yeast *Yarrowia lipolytica* for high-level resveratrol production. Metab Eng.

[50] Seletzky JM, Noak U, Fricke J, Welk E, Eberhard W, Knocke C, Büchs J (2007). Scale-up from shake flasks to fermenters in batch and continuous mode with *Corynebacterium glutamicum* on lactic acid based on oxygen transfer and pH. Biotechnol Bioeng.

[51] Sturmberger L, Menczik P, Moser S, Kotz D, Aleschko M. Scale-dependent effect of helper factor co-expression in Pichia pastoris, In: ICY15 Meets ICYGMB30, Vienna, Austria. 2021, p. B10.

[52] Totaro D, Radoman B, Schmelzer B, Rothbauer M, Steiger MG, Mayr T, Sauer M, Ertl P, Mattanovich D (2021). Microscale perfusion-based cultivation for pichia pastoris clone screening enables accelerated and optimized recombinant protein production processes. Biotechnol J.

[53] Totaro D, Rothbauer M, Steiger MG, Mayr T, Wang HY, Lin YS, Sauer M, Altvater M, Ertl P, Mattanovich D (2020). Downscaling screening cultures in a multifunctional bioreactor array-on-a-chip for speeding up optimization of yeast-based lactic acid bioproduction. Biotechnol Bioeng.

[54] Trassaert M, Vandermies M, Carly F, Denies O, Thomas S, Fickers P, Nicaud JM (2017). New inducible promoter for gene expression and synthetic biology in *Yarrowia lipolytica*. Microb Cell Fact.

[55] Vidal L, Lebrun E, Park YK, Mottet G, Nicaud JM (2023). Bidirectional hybrid erythritol-inducible promoter for synthetic biology in *Yarrowia lipolytica*. Microb Cell Fact.

[56] Wackett LP (2021). Why Is the Biodegradation of Polyfluorinated Compounds So Rare?. mSphere.

[57] Wagner JM, Liu L, Yuan S-F, Venkataraman MV, Abate AR, Alper HS (2018). A comparative analysis of single cell and droplet-based FACS for improving production phenotypes: riboflavin overproduction in *Yarrowia lipolytica*. Metab Eng.

[58] Warringer J, Blomberg A (2003). Automated screening in environmental arrays allows analysis of quantitative phenotypic profiles in *Saccharomyces cerevisiae*. Yeast.

[59] Wittmann C, Schütz V, John G, Heinzle E (2004). Quantification of oxygen transfer in test tubes by integrated optical sensing. J Microbiol Biotechnol.

[60] Yu S, Zhang G, Liu Q, Zhuang Y, Dai Z, Xia J (2023). Construction and testing of *Yarrowia lipolytica* recombinant protein expression chassis cells based on the high-throughput screening and secretome. Microb Cell Fact.

[61] Zagnoni M, Cooper JM (2011). Droplet microfluidics for high-throughput analysis of cells and particles. Methods Cell Bio.

[62] Zhang Q, Wang T, Zhou Q, Zhang P, Gong Y, Gou H, Xu J, Ma B (2017). Development of a facile droplet-based single-cell isolation platform for cultivation and genomic analysis in microorganisms. Sci Rep.

