# Supplementary material for: ‘Small volume—big problem’: culturing Yarrowia lipolytica in high-throughput micro-formats
Source: Microb Cell Fact. 2024 Jun 24;23:184. doi: 10.1186/s12934-024-02465-3 (PMC11197222; doi:10.1186/s12934-024-02465-3)
Supplement: Supplementary file 1 — Additional file 1. [file 12934_2024_2465_MOESM1_ESM.docx]

| System | rpm | Shaking amplitude  [mm] | Culture volume | Δ culture volume [%]  Δ system | **Δ Oxygen transfer rate**  **[kLa] or [OTV]** | reference |
| --- | --- | --- | --- | --- | --- | --- |
| O_96-well | 400 | NA | 200 μL | -30% | **+85%** | (Hermann et al., 2003) |
| O_96-well | 400 | NA | 140 μL |  |  |  |
| O_96-well | 500 | NA | 200 μL | -30% | **+75%** | (Hermann et al., 2003) |
| O_96-well | 500 | NA | 140 μL |  |  |  |
| O_96-well | 450 | 2 | 200 μL | -60% | **+120%** | (Celińska and Gorczyca, 2024) |
| O_96-well | 450 | 2 | 80 μL |  |  |  |
| O_48-well | 450 | 2 | 300 μL | -40% | **+63%** | (Kensy et al., 2005) |
| O_48-well | 450 | 2 | 500 μL |  |  |  |
| O_48-well | 450 | 2 | 200 μL | -60% | **+93%** | (Celińska and Gorczyca, 2024) |
| O_48-well | 450 | 2 | 500 μL |  |  |  |
| O_24-well | 380 | 3 | NA | Δ system | **+9%** | (Doig et al., 2005) |
| O_96-well | 450 | 3 | NA |  |  |  |
| O_24-well | 380 | 2 | 1000 μL | Δ system | **+2%** | (Celińska and Gorczyca, 2024) |
| O_96-well | 450 | 2 | 200 μL |  |  |  |
| □_24-well | 300 | 25 | 2.5 mL | Δ system | **0%** | (Duetz et al., 2000) |
| flask | 300 | 25 | 25 mL |  |  |  |
| □_24-well | 250 | 19 | 2.5 mL | Δ system | **+103%** | (Celińska and Gorczyca, 2024) |
| flask | 180 | 20 | 25 mL |  |  |  |
| □_24-well | 250 | 19 | 2.5 mL | Δ system | **+2%** | (Celińska and Gorczyca, 2024) |
| flask | 180 | 20 | 58 mL |  |  |  |
| Test tube | 300 | 25 | 3 mL | Δ system | **-46%** | (Wittmann et al., 2004) |
| O_96-well | NA | NA | NA |  |  |  |
| Test tube | 180 | 10 | 2 mL | Δ system | **-54%** | (Celińska and Gorczyca, 2024) |
| O_96-well | 450 | 2 | 180 uL |  |  |  |

Table S1. Impact of culture volume change or culturing system format on the relative change in oxygen availability. (NA – information not available; OTV – oxygen transfer velocity)
